# The invasive Red-vented bulbul (*Pycnonotus cafer*) outcompetes native birds in a tropical biodiversity hotspot

**DOI:** 10.1371/journal.pone.0192249

**Published:** 2018-02-01

**Authors:** Martin Thibault, Eric Vidal, Murray Alan Potter, Thierry Sanchez, Fabrice Brescia

**Affiliations:** 1 Institut Agronomique Néo-Calédonien (IAC), Equipe ARBOREAL (AgricultuRE BiOdiversité Et vAlorisation), Païta, New Caledonia; 2 Wildlife and Ecology Group, School of Agriculture and Environment, Massey University, Palmerston North, New Zealand; 3 Institut Méditerranéen de Biodiversité et d’Ecologie marine et continentale (IMBE), Aix Marseille Université, CNRS, IRD, Avignon Université, Centre IRD Nouméa, Nouméa, New Caledonia; 4 Société Calédonienne d’Ornithologie, Nouméa, New Caledonia; University of Auckland, NEW ZEALAND

## Abstract

Invasive alien species are a major cause of biodiversity loss globally, but especially on islands where high species richness and levels of endemism accentuate their impacts. The Red vented bulbul (*Pycnonotus cafer*), a tropical passerine bird that has been introduced widely across locations of high conservation value, is considered an extreme pest. It is currently expanding its range in New Caledonia, one of the world’s biodiversity hotspots. Decisive recommendations on management strategies are required urgently to inform local managers and policy makers, but they should be based on quantitative local evidence, not just on expert opinion. The Red-vented bulbul is widely blamed for its impacts on biodiversity, especially through competition. We used data from 2,472 point counts to explore the abundance relationships between the Red-vented bulbul and 14 other species of bird. Our results revealed a negative relationship between the occurrence of the bulbul and the mean abundance of nine species, all native (or endemic, n = 3) to the New Caledonia archipelago. In contrast, the abundance of other introduced species such as *Acridotheres tristis* (Common myna), *Passer domesticus* (House sparrow) and *Spilopelia chinensis* (Spotted dove) were not affected by the Red-vented bulbul. Moreover, temporal trends in the abundance of impacted species suggest that the Red-vented bulbul may cause niche contractions rather than mortality for native species in man-modified habitats. Monitoring and control of the Red-vented bulbul is recommended to prevent on-going impacts on native bird communities throughout New Caledonia, and its impact on native bird communities elsewhere should be quantified.

## Introduction

Exotic species play a major role in the decline of native species globally, but especially on islands with high species richness, high levels of endemism, and naivety towards novel predators and competitors [1–5]. Defining the appropriate attitude to hold toward introduced species is a matter of debate [6–9]. However, the continuous increase in numbers of alien species across diverse habitats is a reality [10], and there appears to be consensus that field studies and local assessment of their negative impacts are essential to design better responses to biological invasions [11–12]. Recent models suggest that 16% of biodiversity hotspots are highly vulnerable to invasive species [13–15], because of various pressures on native biodiversity through predation, competition, disease transmission, hybridization and ecosystem perturbation [16–18]. Most data on animal invasions have been derived from studies on established and stable alien populations, often from a macro-ecological perspective [19], and interest from researchers and managers has been biased towards some invasive taxa such as mammals [20]. For invasive birds particularly, more data are needed on early-stage dispersal processes and impacts to help predict, prevent, and manage harmful impacts [21–22].

The Red-vented bulbul (RVB), *Pycnonotus cafer* Linnaeus, 1766, is a good example of a species that is currently considered to be a major invasive species [13], more through expert opinion than through scientific assessment of its impacts [23–24]. This species is a tropical passerine from southern Asia that was widely transported as a caged bird from the early 1900s onwards [25]. Several release and escape events led to its successful establishment in at least 36 locations out of 46 where it was introduced, including 27 islands, two continental islands and seven continental areas [24]. Its diet of fruits [26] and its aggressive interspecific behavior [27] are blamed for the damage it causes to crops [28] and its ability to out compete native avifauna [29]. Moreover, its impacts are thought to overlap considerably with other widespread invasive species such as the Common myna (*Acridotheres tristis*) or the Black rat (*Rattus rattus*), and could represent an additive pressure on species of high conservation values [11, 30]. Direct assessment of local impacts and invasion mechanisms for the RVB are scarce [23]. Some authors have claimed that alien populations of the RVB in tropical islands are harmless (Fiji; [31]) whereas others claim that this species should be at the top of invasive species priority lists [29–30].

In New Caledonia, some caged RVBs were released in the capital (Nouméa) around 1983 [32] and the species is now in the “spread” phase [33–34]. The rate of the species range expansion in the main island has increased progressively since its introduction and its range now extends nearly 100 km beyond its initial release site. New Caledonia is a biodiversity hotspot [35], with nearly 60% of its 90 species of terrestrial breeding birds being endemic [36]. With increasing urbanization and habitat transformation, along with the deleterious consequences of mining activities, the additive pressures from invasive species may impact severely upon the conservation of already weakened native bird communities. Therefore, concern amongst managers and scientists about the spread of the RVB has increased over the last decade and the RVB is now considered in law to be a priority pest species in the two provinces of the main island [37–38]. Of particular concern is its supposed contribution to the local decline in some native passerine species in man-modified habitats, but no robust evidence exists that could corroborate or refute this concern.

Here, we studied the impacts of ongoing range expansion of the RVB on terrestrial birds in New Caledonia. The objectives were to i) describe how anthropized habitats shape the early dispersal of this introduced species, ii) identify bird species that may decrease in abundance following the arrival of the RVB, and iii) determine whether an increase in local abundances of the RVB contribute to a decline in native bird species. The results are of relevance to managers at both local and global scales, providing insight into the risks associated with this invasive species. Implications for adapted management strategies are discussed.

## Methods

### Temporal monitoring of terrestrial birds

**Fig 1:**
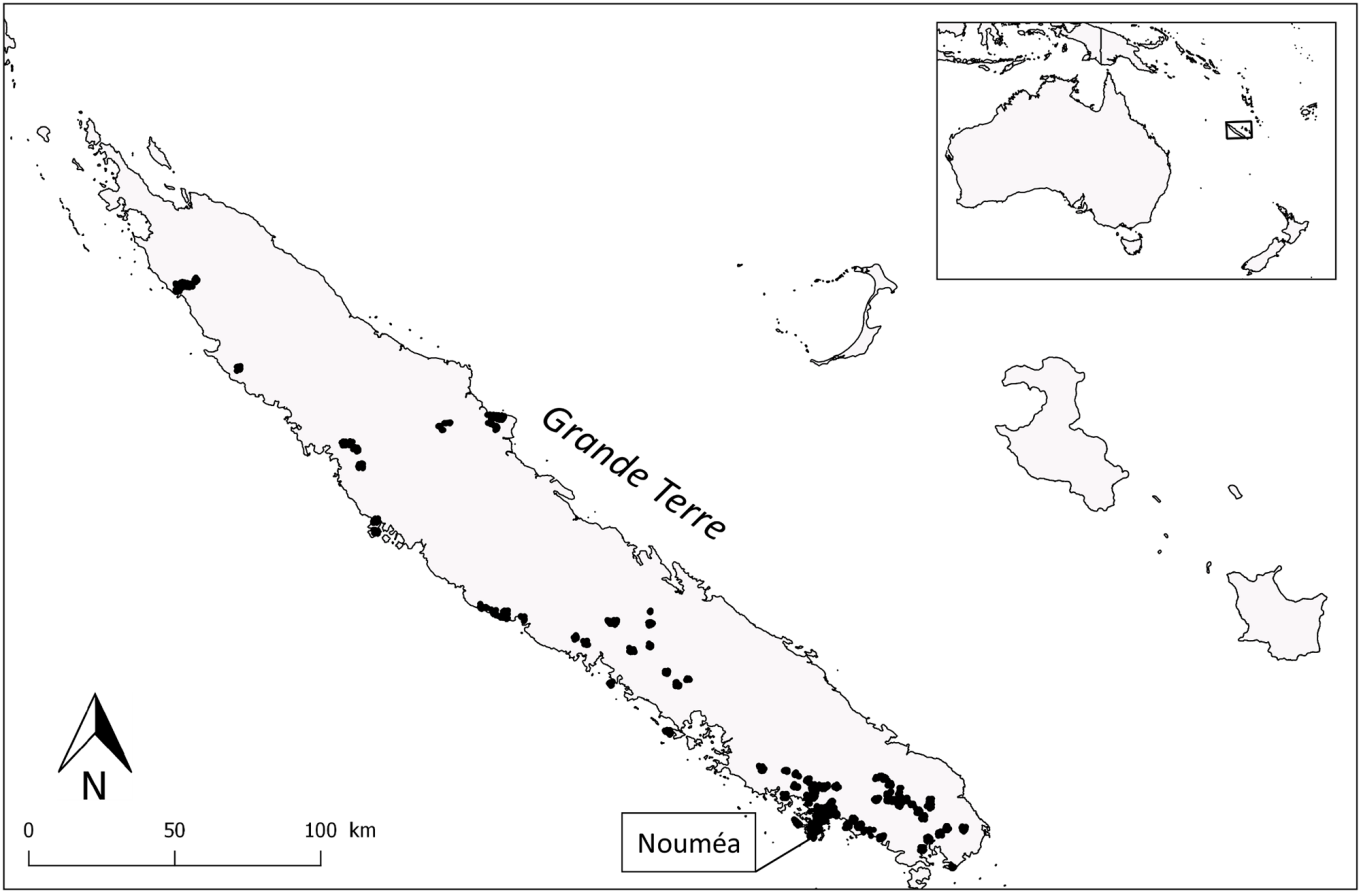
Distribution of stations considered in the monitoring of terrestrial breeding birds in New Caledonia. Points represent sampling stations and correspond to 10 point counts.

Point-count data, as classically used for the temporal monitoring of terrestrial birds [39], were collected on the Grande Terre island from 97 monitoring stations corresponding to 2 478 point counts over six consecutive years (Fig 1, *samplings per year are detailed in next section*). Observers were responsible for the monitoring of a station (2 km^2^) that was covered with 10 randomly distributed listening points spaced a minimum of 250 m apart. The 10 points were monitored in a single day, annually, between October and December. Point counts started 30 minutes after sunrise (range 05.30–06.00 h) and ended at 10.00 h. At each point, the observer waited 3 minutes to avoid any impact of their arrival on bird detection, and then counted every bird heard or seen during a 5-minute period [39].

Because part of the data came from participative monitoring, and because the location of some points evolved together with habitat perturbations, the sampling effort was not homogeneous over the monitoring period. We started the monitoring with 77 points in 2010, sampled 309 and 391 points in 2011 and 2012 respectively, and reached more than 610 points in 2013 and 2014 before a small decrease in 2015 to 470 sampled points. However, the year of sampling was not found to be a significant source of variation for bird abundance in any regression or generalized mixed models analysis. Most of the sampled sites were located within or next to the current distribution range of the RVB.

Point count data were extracted for 15 bird species including the RVB. Ten species were selected because they were passeriforms of similar size to the RVB (less than 20 cm in height) and shared the same habitat (sparse secondary forest, scrub, orchards and gardens). We considered the Fan-tailed gerygone (*Gerygone flavolateralis*), the Long-tailed triller (*Lalage leucopyga*), the Grey-eared honeyeater (*Lichmera incana*), the Melanesian flycatcher (*Myiagra caledonica*), the New Caledonian myzomela (*Myzomela caledonica*), the Rufous whistler (*Pachycephala rufiventris*), the House sparrow (*Passer domesticus*), the Grey fantail (*Rhipidura albiscapa*), and two silvereyes species (*Zosterops xanthochroa and Z*. *lateralis*). The two silvereyes species are difficult to distinguish in the field, so were considered here as a single taxon. Two species of a larger size were frequently observed feeding on the same tree species as the RVB: the Spotted dove (*Spilopelia chinensis*) and the Coconut lorikeet (*Trichoglossus haematodus*), and were also considered in our analysis. We added two larger species with restricted distribution status that often responded to RVB calls, the New Caledonian crow (*Corvus moneduloides*) and the New Caledonian friarbird (*Philemon diemensis*). Finally, the Common myna (*Acridotheres tristis*) was included to test for potential confounding effects of the RVB and this other widespread and abundant invasive species. Eleven of the selected species were native to New Caledonia; the other three were introduced species. The full species list and their corresponding origin and conservation status are given in S1 Table.

### Environmental data

On each sampling occasion the GPS location, date, and habitat type were recorded. Habitat was characterized by two factors. First, by one of six pre-selected macro-habitat types: 1) aquatic; 2) forest; 3) mining maquis; 4) shrubland; 5) agricultural areas; and 6) inhabited areas; second, four-to-six more precise descriptors of the habitat. The full list of habitats considered is presented in S2 Table. Mapping of the presence of the RVB in this dataset revealed that this species was concentrated in man-modified areas (around 40% of sampled points) and was very scarce in other habitats (<10% of points each). We therefore focused our analyses on the 579 point counts located in inhabited habitat (category 6) to avoid spatial autocorrelation. Determination of the impacts of the RVB on other species required ‘control’ locations from which the RVB is currently absent. For these, we selected man-modified locations outside of the RVB’s current range but where the other 15 targeted species were present (11<N<329; depending on the species). Using GPS data, we calculated the distance between each sampling point and the location in Nouméa where RVBs are supposed to have been originally released [32].

### Data analysis

All statistical analyzes were conducted using the R software, version 3.3.2 [40]. To describe the early dispersal of the RVB, we ran a generalized linear mixed effect model with the RVB’ abundance as a quantitative variable explained by habitat, year and distance to introduction point. Then, we explored the relationships between the RVB’ presence and abundances of local birds through pairwise T-tests of their mean abundance, depending on whether the RVB was present or not. In the last step we selected four native species that lived sympatrically with the RVB (present at the same point during the same sampling session) and used model selection and model averaging techniques to explore RVB abundance as an explanatory variable of the abundances of these native species. Relationships were considered significant when p_values were <0.01.

To test which environmental factors affected RVB abundance, we used a generalized linear mixed model from the package “lme4” v.1.1–7 [41] that considered ‘site’ as a random factor. We used a subset of the data that considered only the points located inside the RVB’s current range. In this model, distance to the introduction point was used as a fixed effect corresponding to the density gradient. Macro-Habitat (S2 Table) was used as a factor to account for the RVB’s tight association with man-modified areas, and year was also used as a numeric fixed effect to account for an expected increase in RVB abundance over the invasion process. Bootstrapped confidence intervals were set at 98% of confidence level. Confidence intervals were calculated from 500 parametric bootstrap simulations.

We compared the mean abundance of the 15 bird species in the presence or absence of the RVB using a series of parametric Students t.test. This test allows a comparison between two subgroups from a quantitative variable. We compared the abundance of each species when the RVB was present or not, by running 14 tests using the 14 data subsets corresponding to each target species. To conduct adapted analyzes for each targeted species, we calculated the range overlap between each of these and that of the RVB. For this we first plotted the Maximum Convex Polygone (MCP) including all presence points for each of the 15 species, using the package “adehabitatHR” [42], and then calculated the percentage of overlap using “rgeos” [43]. The number of co-occurrence points was calculated and used as an index of mixed model “feasibility” for further analyzes.

To explore the mechanisms leading to a decrease in the abundance of bird species, we selected “impacted” species that were observed 30 times or more in sympatry with the RVB. The relationships between abundance of these birds and RVB abundance, abundance of *A*. *tristis*, sub-habitat, and year were investigated using a Poisson Log generalized linear mixed model [44], which included ‘site’ as a random effect. Abundance of the RVB and sub level of “inhabited areas” habitat were the main explanatory variables. Abundance of the introduced *A*. *tristis* was used as a fixed effect in full models to account for any habitat partitioning with the RVB [43]. Year was used as a fixed effect to account for a potential temporal autocorrelation in our dataset. Usefulness of the random effect was tested via ANOVA between full models. We modeled the abundance of the species using the package “lme4” v.1.1–7 [45] and checked graphically the correctness of the error variance [46]. We then performed a model selection using a dredging procedure, selecting and averaging all models that were within 2 AICc units of the most parsimonious model (i.e., the lowest AICc). This was done using the package “MuMIN” [47]. The MuMIn package does not allow random effect averaging [47], so estimates and confidence intervals for random effects were calculated from the best model only. We controlled for the potential influence of time in the abundance of bird species by plotting the abundance of the main species over the monitoring period in man-modified habitats. Results are given in estimate per factor and bootstrapped confidence intervals at 98% of confidence level. Confidence intervals were calculated from 200 parametric bootstrap simulations.

## Results

### Red vented bulbul’s distribution

**Fig 2:**
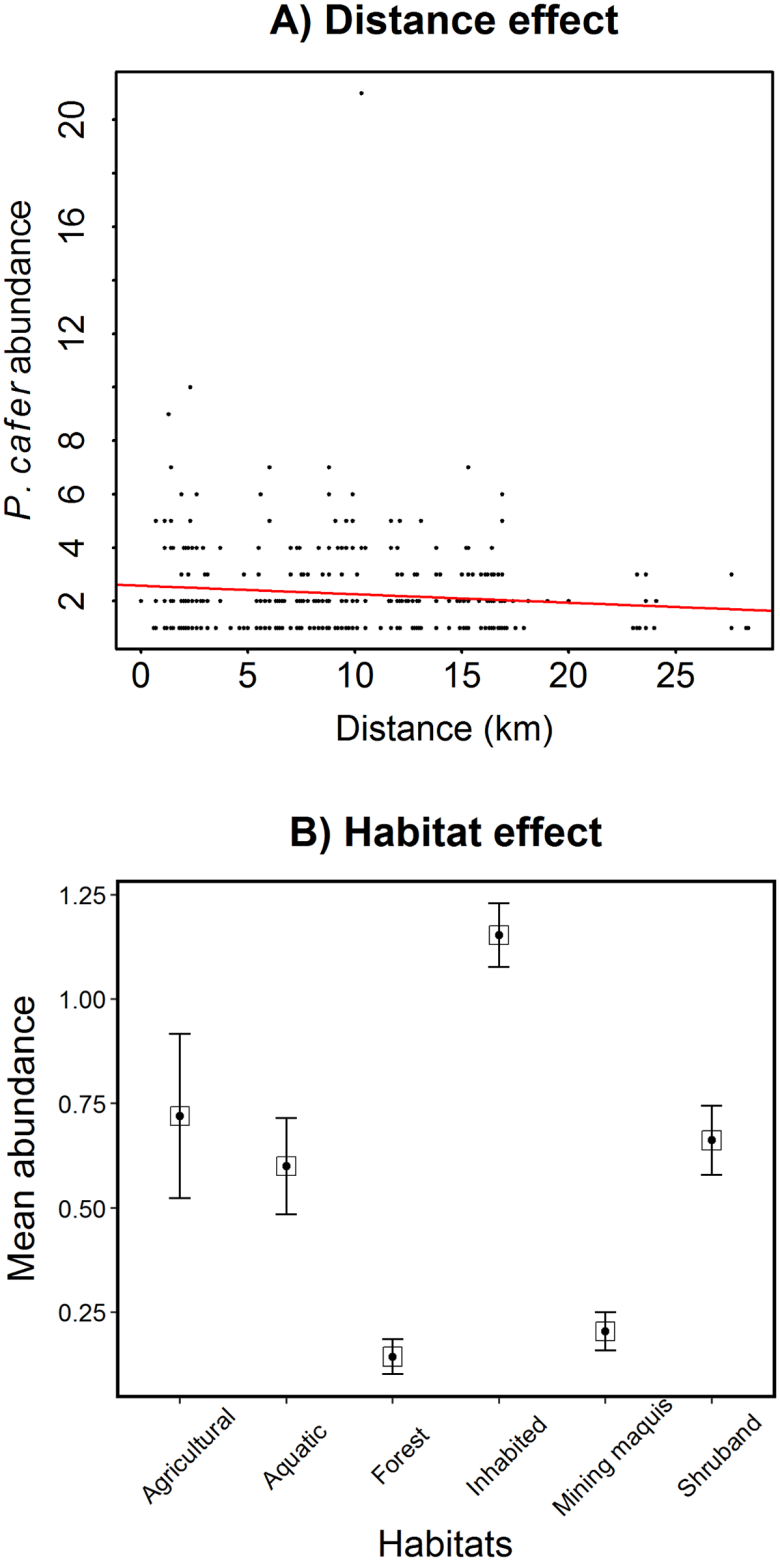
Relationship between the abundance of the RVB and a) the distance from the introduction point in Nouméa and b) the category of habitat sampled. Bars in the Fig 2b represent the standard errors of the means.

The RVB was detected and counted at 346 points, mainly concentrated around Nouméa (the initial introduction location). In 2015 the RVB was commonly present in all sites within 30 km of the original introduction site in Nouméa. Based on all point counts available from this area (N = 1139), we found that the RVB mean abundance did not vary significantly across years (S3 Table). The average numbers of RVBs per point were 1.02 ± 0.17 (*n* = 58) and 0.42 ± 0.07 (*n* = 184) in 2010 and 2015 respectively. The mean number of RVBs was significantly higher in man-modified habitats (1.19 ± 0.09; *n* = 216) and lower in forest habitats (0.14 ± 0.04; *n* = 444). We also found a negative relationship between RVB abundance and distance from the historic introduction site (-0.08 ± 0.02; P< 0.001; Fig 2).

### Impact of Red-vented bulbul presence on the abundance of other bird species

**Fig 3:**
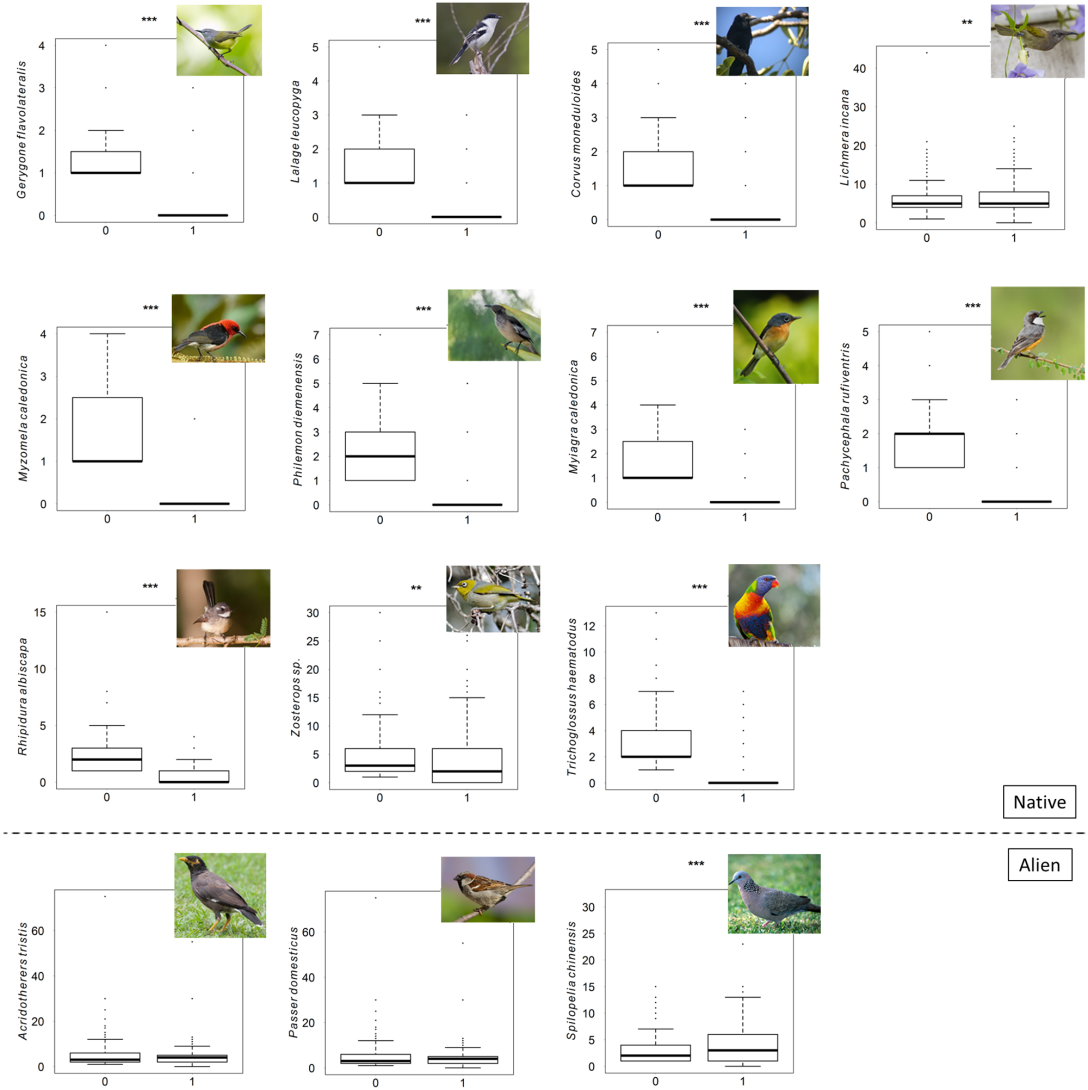
Mean abundance of 14 bird species in man-modified habitats depending on the presence of the RVB. Mean abundance: average number of individuals per point. Significant tests are marked with *** (99% confidence) and ** (95% confidence). *0 = absence; 1 = presence*.

We found a negative relationship between the presence of the RVB and the abundance of nine of the 14 bird species considered in man-modified habitats (Fig 3), namely the *C*. *Moneduloides*, *G*. *flavolateralis*, *L*. *leucopyga*, *M*. *caledonica M*. *caledonica P*. *rufiventris*, *P*. *diemensis*, *R*. *albiscapa*, and *T*. *hematodus*. The distribution of each of the 14 bird species overlapped with at least 65% of the current distribution range of the RVB (S4 Table). The abundance of only one species, the spotted dove, appeared to be higher when the RVB was present (t: 2.84; *p* = 0.0048; S4 Table). The mean abundances of two introduced species, *A*. *tristis* and *P*. *domesticus* did not differ significantly when the RVB was present (4.4 ± 0.36 and 6.61 ± 0.77; *n* = 215) or absent from the point (4.72 ± 0.36; *n* = 261 and 7.99 ± 0.82; *n* = 144, S4 Table). There was also no significant effect of whether RVBs were present or absent on *L*. *incana* or the silvereyes at the 0.01 threshold (t: 1.73; *p* = 0.083 and t: -1.7; *p* = 0.088).

### Effect of Red-vented bulbul abundance on the reduction of local bird populations

**Fig 4:**
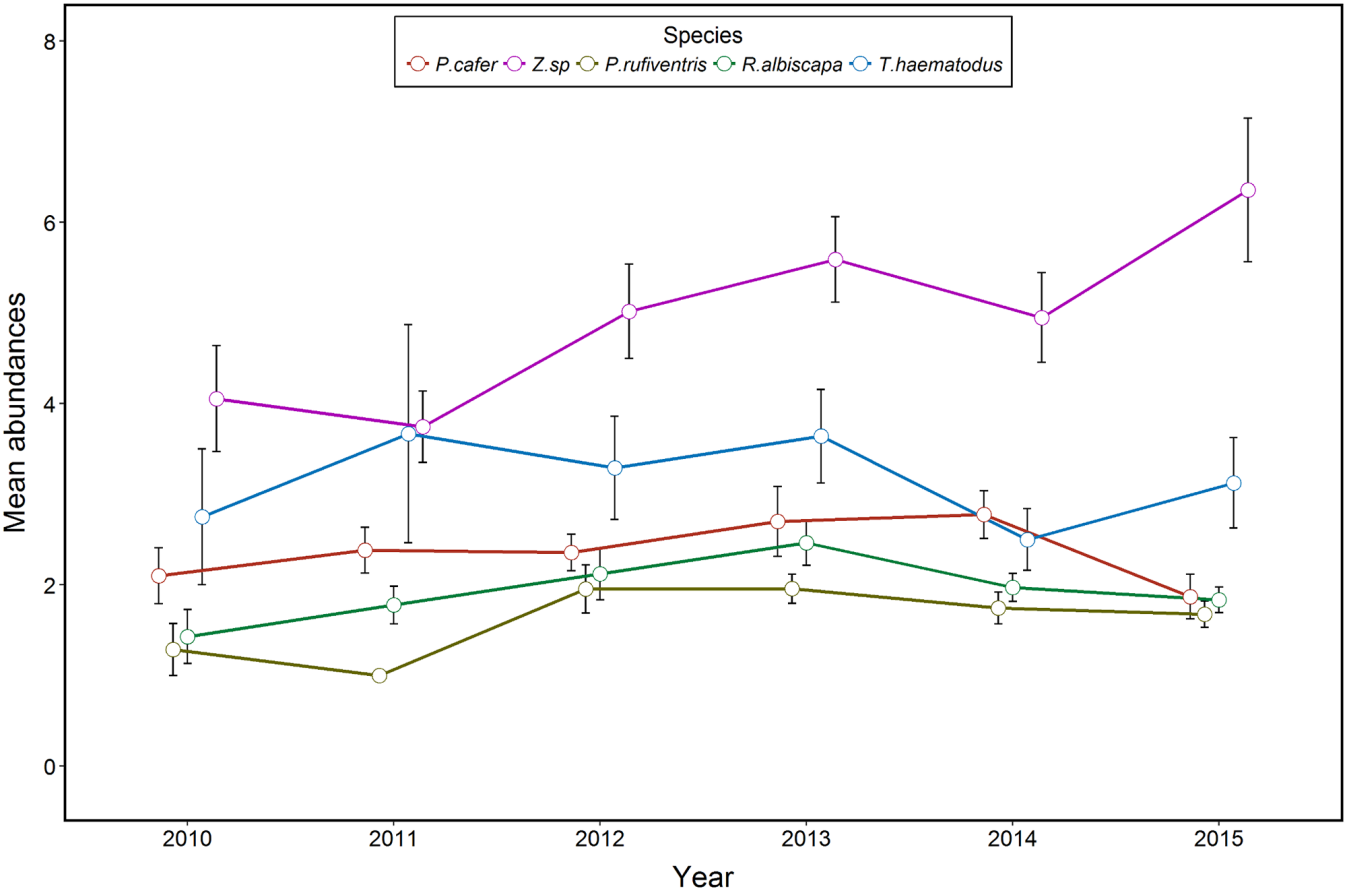
Trends through time in the abundance of *Pycnonotus cafer*, *Zosterops* spp., *Pachycephala rufiventris*, *Rhipidura albiscapa* and *Trichoglossus haematodus* in man-modified habitat over time during the monitoring of terrestrial breeding birds. Mean abundance: average number of individuals per point. The increase in the abundance of *Zosterops* spp. is significant (see Table 1).

**Fig 5:**
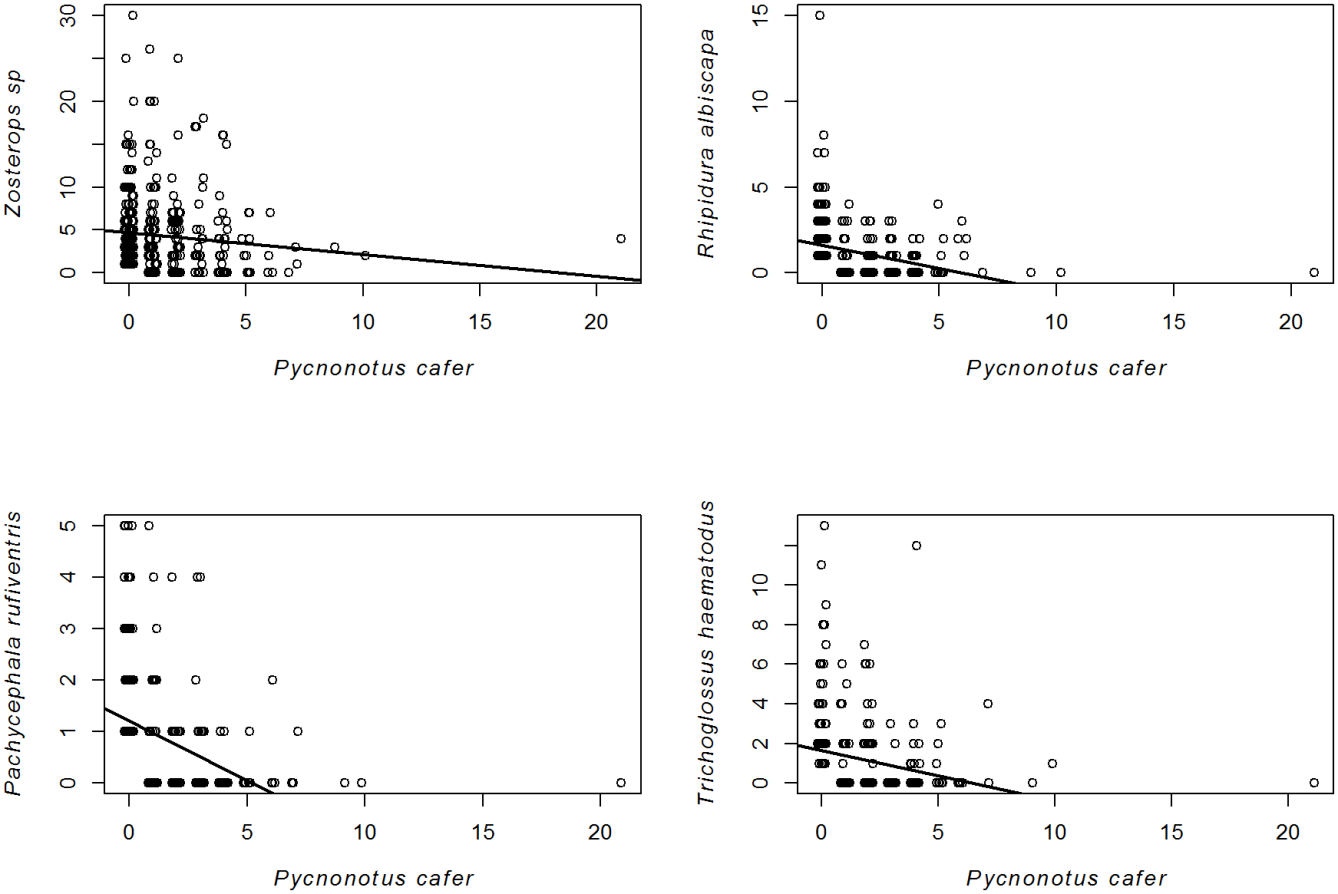
Abundance relationship between the RVB and four bird species in man-modified habitats. Mean abundance: average number of individuals per point.

Among the nine bird species that were less abundant when co-occurring with the RVB, four species (*R*. *albiscapa*, *Z*. *spp*, *P*. *rufiventris* and *T*. *haematodus*) shared more than 30 locations with the RVB (S4 Table). Table 1 presents the estimates and confidence intervals for each parameter that was kept through the model selection process. Results of model selection and averaging for the abundance are detailed in S5 Table. Time (year) was never associated with a decrease in bird abundances, but contributed significantly to explain an overall increasing abundance of silvereyes in urban habitats (Fig 4). Different degrees of urbanization contributed to explain the abundance of the *R*. *albiscapa* and *P*. *rufiventris* (Table 1), with more individuals of these species being counted in rural habitats (2.37 ± 0.27; *n* = 71 and 1.54 ± 0.18; *n* = 54) than in urban habitats (0.44 ± 0.06; *n* = 155 and 0.26 ± 0.06; *n* = 141). We found a negative relationship between the abundance of the RVB in man-modified habitats and that of the four species (Fig 5). The abundance of *A*. *tristis* did not contribute to explain variations in the abundance of the four species.

**Table 1:** Parameter estimates and confidence intervals of the averaged models explaining the abundance of four bird species in man-modified habitats. Mean abundance: average number of individuals per point. Significant parameters are in bold.

	*Rhipidura albiscapa*	*Zosterops spp*.	*Pachycephala rufiventris*	*Trichoglossus haematodus*
	Estimates [98% CI]	Estimates [98% CI]	Estimates [98% CI]	Estimates [98% CI]
Random effect				
**σ**_*(Site)*_	0.14[0.13;0.53]	0.36[0.41;0.77]	0.13[1.57E-5; 0.54]	0.71[0.49;1.14]
Fixed effects				
*Intercept*	-0.26[-0.73;0.21]	**0.67[0.30;1.04]**	-0.76[-1.48;-0.05]	0.05[-0.57;0.68]
*Actri*	-0.03[-0.07;0.01]	0.01[00.04 E-1;0.02]	-0.03[-0.08;0.02]	-0.05[-0.16;0.07]
*sHabitat*_*[suburban]*_	**0.84[0.39;1.28]**	-0.21[-0.46;0.04]	**1.10[0.50;1.70]**	--
*sHabitat*_*[rural]*_	**1.10[0.62;1.59]**	-0.24[-0.60;0.12]	**1.29[0.69;1.90]**	--
*sHabitat*_*[tribal]*_	**1.01[0.39;1.62]**	-0.01[-0.50;0.47]	**1.12[0.37;1.88]**	--
*Pycaf*	**-0.28[-0.39;-0.16]**	**-0.08[-0.12;-0.04]**	**-0.43[-0.61;-0.25]**	**-0.27[-0.38;-0.16]**
*Year*	0.02[-0.07;0.11]	**0.10[0.06;0.15]**	0.06[-0.07;0.18]	0.08[-0.02;0.17]

## Discussion

The RVB is currently expanding its range over the main island of New Caledonia, so an abundance gradient from the point of first introduction outwards was expected [33]. Alien species introductions in capital cities such as Nouméa is common in island territories [48] and it may favor alien species that are able to cope with close proximity to humans [49–50]. Because of traffic, cities are suspected to foster the invasion process toward rural habitats [51]. This is exactly what we found here, with RVBs being most predominant in man-modified habitats. The RVB is often located along roads and in gardens where plant species from its native range are present [26].

Our analysis indicates that nine of the 14 bird species monitored in man-modified habitats were less abundant when the RVB was present. The response of local birds to the spread of the RVB is therefore of great concern since 55 of the 90 breeding species of terrestrial birds in New-Caledonia are strictly endemic [36]. Fifty-four of them are legally protected in the Southern Province [38]. Eight of the species that declined in abundance in the presence of the RVB were passeriform and one was a Psittaciformes. The other species were significantly less abundant at sample points occupied by the RVB and shared more than 30 locations with the invader. The abundance of only two native bird species appeared to be unaffected by the presence of the RVB. *L*. *incana* remains very common in man-modified habitats and field observations suggest that this species is undisturbed in the presence of the RVBs or their calls. It is a very active, noisy, gregarious honeyeater [52]. The second unaffected native taxon was *Zosterops spp*.

Our abundance models confirmed most of the findings from the first analysis and provided additional details on interspecific competitive relationships. Interestingly, abundance models detected a negative effect of the RVB on *Z*osterops *spp* in man-modified habitats that our point-sampling data had failed to detect. The GLM analyses identified an increase in the mean abundance of *Zosterops spp* in human modified habitats through time, which appeared to be independent of the presence or absence of the RVB. This likely obscured detection of a negative impact of the RVB on silvereyes in our point-count dataset.

We found negative abundance relationships between the RVB and the three other species that we tested. Abundance of *R*. *albiscapa* and *P*. *rufiventris* also showed an urbanization gradient, with more birds detected in rural than in urban and suburban habitats. Conversely, abundance of the RVB was the main factor explaining variations in the mean abundance of *T*. *haematodus*. The three passerine species share several life history traits with the RVB including similar length (approx. 16 cm), nesting height (2–3 m), nest structure (cup-like shape), incubation period (14 days) or clutch size (3±1) [53]. On the other hand, *R*. *albiscapa* and *P*. *rufiventris* are considered mainly insectivorous [54] and should not compete with the RVB for food, as the RVB diet comprises mainly fruit [55]. We believe that native passerine species may suffer from the foraging behavior of the RVB, which is known to be active and aggressive toward other species in its preferred forage trees [27,30,56]. Such negative interaction due to aggressive behavior has already been demonstrated in the Noisy minor *Minorina melanocephala* [57]. *T*. *haematodus* is a generalist bird that feeds mostly on fleshy fruits, nectar and pollen [58]. Like the RVB, it is highly mobile and able to forage over a large area. Thus, our result could reflect a shift in forage tree selection by *T*. *haematodus* when too many RVBs are present.

None of the alien species considered in this study (*A*. *tristis*, *P*. *domesticus*, *S*. *chinensis*) were less abundant when the RVB was present. This is partly consistent with the finding of Bates et al. (2014) [43] on the foraging relationship between the RVB and *A*. *tristis* in Moorea, French Polynesia. Those authors suggested that the two invasive species were able to coexist in the same habitat without competing for food resources thanks to different foraging strategies. Such partitioning could also apply to *P*. *domesticus* and *S*. *chinensis*, as the two species are more granivorous and ground-foraging than the RVB [59–60]. Moreover, the three species were widely introduced and are abundant in cities worldwide [61–63]. Time partitioning in the use of the same resource could allow an alien population of RVB to expand, even in locations occupied by other invasive bird species [43]. Such neutral interaction between sympatric invasive species is the basis for additive impacts on resources and native competitors that can lead to major conservation issues [64].

From a conservation perspective, our data raise concerns about the negative effects of RVB establishment on the abundance of native birds. Our results do not reflect a temporal trend in bird abundance, as none of the native species considered here showed a decline in abundance over the monitoring period in man-modified habitats. We believe that the RVB may drive a reassembly of native species toward sub-optimal locations along an urban-rural gradient, as suggested in the niche reduction hypothesis [65]. This is consistent with recent assessments on the effects of urbanization on the distribution of a number of other bird species [66]. These authors suggest that urbanization promotes the establishment and development of species that can cope with human activities. These species are often generalists and more competitive in urban habitats [50,67]. Urbanization tends to drive a reassembly of bird species along an urbanization gradient through competitive interactions, with native species being more competitive in rural habitats and alien bird species more successful in urban centers [68]. In 1979, Watling suggested this process as an explanation for the habitat shift in native birds of Fiji following the establishment of the RVB there [31]. Our results confirm that the RVB appears able to coexist with other alien birds in man-modified habitats via habitat partitioning [43]. However, despite this partitioning, the RVB may negatively affect the distribution and abundance of native birds in urban habitats through its aggressive interspecific behavior enabling it to out-compete native species and dominate access to food resources.

An increase in mean abundance of the RVB was expected over the course of our study due to the population expansion process, but this was not significant in our models. This suggests that time may not be a major driver of RVB abundance at a specific location, and that habitat characteristics and distance to the core of the local range are of greater importance. Establishment of the RVB in New Caledonia is recent and we conducted this study at an early dispersal stage. The temporal monitoring of terrestrial birds of New Caledonia has started in 2010, and data on birdabundance in the current range of the RVB before its establishment are lacking. So, it is very difficult to conclude on a temporal pattern in the effect of RVB on the abundance of native bird species. However, invasion by the RVB is still ongoing in New Caledonia. Here, we suggest to benefit from this original context by using spatial patterns as proxies of temporal ones, using inhabited areas that have not been invaded as background data. The hypothesis we formulated should be confirmed by measuring directly changes in bird abundances at sites that became invaded after 2010. However, since long-term temporal monitoring of birds is time consuming, required funding and consequent human resources, such data are often lacking in small territories. Where background data are lacking, we believe that the method used here could be of value while assessing threats from introduced species. When trying to implement this kind of long-term monitoring, involvement of volunteer citizens has been demonstrated to be a powerful and efficient tool [69].

Because we focused on a potential effect of the presence of red-vented bulbuls rather than population estimates, we did not calculate the detection probability of the bird species we studied. This could have led to an overestimation of the impact of the RVB on the abundance of local birds if some species called less because of the presence of bulbuls. However, we believe this potential bias to be less important in the inhabited areas we sampled, as there, birds were detected through visual locations more than acoustic ones compared to other habitat contexts. Furthermore, less bird calls in the presence of the RVB could also be considered as a negative impact, and the behavioral pressure of introduced RVB on native birds could contribute to explain a reassembly of the bird community along an urbanization gradient. The respective contribution of competition for trophic resources and behavioral competition could be the subject of further studies on the ecological mechanisms that shape impacts of introduced species.

In New Caledonia, the RVB is currently recorded exclusively in man-modified habitats that are most suitable and favor its dispersal. In its native range, the RVB is abundant in open habitats but is rarely found in mature forest [70]. However, changes in RVB abundance and distribution in Tahiti over a 10-year period have shown that the species can establish in native habitats after an initial population lag-phase in man-modified habitats [30,71]. Therefore, if nothing is done to slow down or stop its dispersal in New Caledonia, the RVB will likely continue its expansion across the New Caledonia archipelago. The negative effects of the RVB at comparatively low densities on native New Caledonian bird species raise concern about escalating impacts as the RVB’s range expands and its densities increase into more native and natural habitats. New Caledonian forests host an important endemic biodiversity of not just birds but also insects and reptiles [72–74] that may be impacted negatively through interaction with the RVB [24]. Predation upon, or competition with, these species could be more damaging than the ones we have identified here because native species living in less disturbed habitats are often particularly predator-naïve and sensitive to perturbation [75–76].

There is an urgent need for dedicated monitoring of the range expansion of the RVB in New Caledonia, and design and implementation of an effective management plan based on research experiments such as exclusion tests. Depending on both social and financial support, eradication programs could be implemented that would minimize further conservation impacts of the RVB in New Caledonia, such as has been achieved through control of invasive *A*. *tristis* in the Seychelles [77–78]. The impact of the RVB elsewhere has been profound, such as its competitive pressure upon the Tahiti monarch, *Pomarea nigra*, in French Polynesia [29], but shooting, trapping and poisoning of RVBs and *A*. *tristis* has been efficient in reducing the impacts of these species on endemic species of birds there [71]. Serious consideration of similar intervention to confine and control the RVB in New Caledonia is required, and its impact on native species throughout its exotic range should be quantified.

## Conclusions

This study generated quantitative data on the impact of invasive Red-vented bulbul on both native and other exotic species of birds in New Caledonia. Competition with native avifauna is one of the three serious impact categories associated with the RVB [24]; the other two being damages to plants [28] and dispersal of noxious plant seeds [79]. This is the first quantitative assessment of the impacts of a RVB invasion on other bird species. Our data indicate that this tropical passerine is better adapted to human-induced ongoing habitat modification on a tropical island than are the native bird species studied here. Presence of the RVB was associated with lower abundances of most native bird species in habitats where it established. This suggests that establishment of the RVB could accentuate the negative impacts of urbanization on native birds around Nouméa and that dispersal of the RVB along urban corridors in New Caledonia may already be causing a shift in the distribution of several native passerine species from the center to the edges of man-modified habitats. Enhanced monitoring of the range expansion of the RVB in New Caledonia and implementation of an effective management plan is required urgently. Whether the RVB is having similar impact on native species elsewhere in its exotic range should be investigated.

## Supporting information

S1 Table List of bird species considered in the study. The Order, Family and species names are given for each studied bird species, as well as the authority reference. The origin of each species is provided regarding their presence in New Caledonia. Local conservation status, based on the Code de l’environnement de la Province Sud (DEPS, 2016), indicates if these species are protected or considered as pest in the Current range of the red-vented bulbul.
(DOCX)

S2 Table Full list of habitats considered in the point counts monitoring. (DOCX)

S3 Table Parameter estimates and confidence intervals from the linear mixed model investigating the distribution of the Red-vented bulbul abundance within its current range. Significant parameters are in bold.
(DOCX)

S4 Table Mean abundance of 14 bird species in man-modified habitats depending on the presence of the Red-vented bulbul. The “RVB overlap” column represent the percentage of the current range of the bulbul where each species is considered present. Sympatry corresponds to the number of sampling points at which the species as been recorded together with the bulbul during the same sampling session. Results of Student’s t tests between the abundance of 14 bird species and the presence of the red-vented bulbul are provided. The “Effect” column indicates if presence of the bulbul affect the abundance of the species in a positive or negative way. Significant results are in bold.
(DOCX)

S5 Table Model selection and averaging on the mean abundance of four species of native New Caledonian birds. Pycaf and actri represent the abundance of *Pycnonotus cafer* and *Acridotheres tristis* respectively. sHab represents sub-habitats. Model selection and averaging was conducted with the abundance of four species as explained variables. Characteristics of models considered in the averaging are described according the four indexes: 1) **K** is number of degree of freedom, 2) **-LL** is the log-likelihood score 3) **AICc** is the corrected Akaike criterion score and 4) **ω** is the weight of each model.
(DOCX)

S1 Data Raw data frame of the 2473 point counts used in this study. Columns “site”, “index”, and “point” describe the location of each point. X and Y are the GPS coordinates in the WGS84 system. “Day” correspond the day number (1–365). The “hab” and “hab1” columns describe respectively the macro-habitat (6 levels) where the point was located, and corresponding habitat sub level. “Dist” correspond to the distance from the historical introduction point.
(CSV)
