# Supplementary material for: The invasive Red-vented bulbul (Pycnonotus cafer) outcompetes native birds in a tropical biodiversity hotspot
Source: PLoS One. 2018 Feb 1;13(2):e0192249. doi: 10.1371/journal.pone.0192249 (PMC5794173; doi:10.1371/journal.pone.0192249)
Supplement: S1 Table — The Order, Family and species names are given for each studied bird species, as well as the authority reference. The origin of each species is provided regarding their presence in New Caledonia. Local conservation status, based on the Code de l’environnement de la Province Sud (DEPS, 2016), indicates if these species are protected or considered as pest in the Current range of the red-vented bulbul. (DOCX) [file pone.0192249.s001.docx]

**S1 Table**. **List of bird species considered in the study.** The order, Family and species names are given for each studied bird species, as well as the authority reference. The origin of each species is provided regarding their presence in New Caledonia. Local conservation status, based on the Code de l’environnement de la Province Sud (DEPS, 2016), indicates if these species are protected or considered as pest in the Current range of the red-vented bulbul.

| Order | Family | Species | Authority | Origin | Local Conservation Status^1^ |
| --- | --- | --- | --- | --- | --- |
| **Columbiforms** | Columbidae | *Spilopelia chinensis* | *Scopoli, 1786* | Alien | Not Evaluated |
| **Passeriforms** | Acanthizidaie | *Gerygone flavolateralis flavolateralis* | *Gray, 1859* | Native | Protected |
|  | Campephagidae | *Lalage leucopyga montrosieri* | Verreaux & Des Murs, 1860 | Native | Protected |
|  | Corvidae | *Corvus moneduloides* | *Lesson, 1831* | Native | Endemic |
|  | Meliphagidae | *Lichmera incana incana* | *Latham, 1790* | Native | Protected |
|  |  | *Myzomela caledonica* | *Forbes, 1879* | Native | Endemic |
|  |  | *Philemon diemenensis* | *Lesson, 1831* | Native | Endemic |
|  | Monarchidae | *Myiagra caledonica caledonica* | *Bonaparte, 1857* | Native | Protected |
|  | Pachycephalidae | *Pachycephala rufiventris xanthetraea* | *Latham 1801* | Native | Protected |
|  | Passeridae | *Passer domesticus* | *Linnaeus, 1758* | Alien | Not Evaluated |
|  | Pycnonotidae | *Pycnonotus cafer* | *Linnaeus, 1766* | Alien | Invasive |
|  | Rhipiduridae | *Rhipidura albiscapa bulgeri* | *Sparrman, 1787* | Native | Protected |
|  | Sturnidae | *Acridotheres tristis* | *Linnaeus, 1766* | Alien | Potentially harmful |
|  | Zosteropidae | *Zosterops sp^2^* | *Vigors & Horsfield, 1827* | Native | Protected |
| **Psittaciforms** | Psitaccidae | *Trichoglossus haematodus* | *Linnaeus, 1771* | Native | Potentially harmful |
|  |  |  |  |  |  |
|  |  |  |  |  |  |
|  | *1: Endemic species are protected* | |  |  |  |
|  | *2: lateralis griseonata + xantrochroa* | |  |  |  |
