# Supplementary material for: The invasive Red-vented bulbul (Pycnonotus cafer) outcompetes native birds in a tropical biodiversity hotspot
Source: PLoS One. 2018 Feb 1;13(2):e0192249. doi: 10.1371/journal.pone.0192249 (PMC5794173; doi:10.1371/journal.pone.0192249)
Supplement: S2 Table — (DOCX) [file pone.0192249.s002.docx]

**S2 Table. Full list of habitats considered in the point counts monitoring.**

|  | Macro-habitats | sub-levels |
| --- | --- | --- |
| 1) | **Aquatic** |  |
|  | i. | Fresh water |
|  | ii. | Sea water |
|  | iii. | Brackish water |
|  | iv. | Mangroves |
|  | v. | Marsh |
| 2) | **Forest** |  |
|  | i. | Dense rainforest |
|  | ii. | Dense rainforest on mining soil |
|  | iii. | Rainforest on limestone |
|  | iv. | Sclerophyll forest |
|  | v. | Other |
| 3) | **Mining maquis** |  |
|  | i. | Bare ground |
|  | ii. | Sparse vegetation |
|  | iii. | Ligno-herbaceous maquis |
|  | iv. | Para-forest maquis |
| 4) | **Thickets, Savannah** |  |
|  | i. | Sparse vegetation |
|  | ii. | Grassland |
|  | iii. | Thickets |
|  | iv. | Littoral vegetation |
|  | v. | Niaouli savannah |
|  | vi. | Niaouli forest |
| 5) | **Agroforest areas** |  |
|  | i. | Bare ground |
|  | ii. | Cultivated area |
|  | iii. | Orchard |
|  | iv. | Forestry plantation |
| 6) | **Inhabited areas** |  |
|  | i. | Urban (city) |
|  | ii. | Suburban (village) |
|  | iii. | Rural |
|  | iv. | Tribal |
