# Supplementary material for: The invasive Red-vented bulbul (Pycnonotus cafer) outcompetes native birds in a tropical biodiversity hotspot
Source: PLoS One. 2018 Feb 1;13(2):e0192249. doi: 10.1371/journal.pone.0192249 (PMC5794173; doi:10.1371/journal.pone.0192249)
Supplement: S3 Table — Significant parameters are in bold. (DOCX) [file pone.0192249.s003.docx]

**S3. Parameter estimates and confidence intervals from the linear mixed model investigating the distribution of the red-vented bulbul abundance within its current range.** Significant parameters are in bold

| Sources of variation |  | Estimates | 98% CIs |
| --- | --- | --- | --- |
| **Random effect** |  |  |  |
|  | **σ***_(Site)_* | 0,88 | [0,58;1,26] |
| **Fixed effects** |  |  |  |
|  | *Intercept* | -0,07 | [1,16; 0,63] |
|  | *Distance to origin* | **-0,08** | **[-0,13;-0,02]** |
|  | *Habitat_[Forest]_* | **-0,82** | **[-1,43;-0,36]** |
|  | *Habitat_[Inhabited]_* | **0,61** | **[0,21;1,07]** |
|  | *Habitat_[maquis]_* | -0,57 | [-1,54;0,24] |
|  | *Habitat_[shrubland]_* | 0,07 | [-0,37;0,67] |
|  | *Habitat_[agricultural]_* | 0,21 | [-1,29;1,11] |
|  | *Year* | -0,04 | [-0,11;0,03] |
