# Supplementary material for: The invasive Red-vented bulbul (Pycnonotus cafer) outcompetes native birds in a tropical biodiversity hotspot
Source: PLoS One. 2018 Feb 1;13(2):e0192249. doi: 10.1371/journal.pone.0192249 (PMC5794173; doi:10.1371/journal.pone.0192249)
Supplement: S4 Table — The “RVB overlap” column represent the percentage of the current range of the bulbul where each species is considered present. Sympatry corresponds to the number of sampling points at which the species as been recorded together with the bulbul during the same sampling session. Results of Student’s t tests between the abundance of 14 bird species and the presence of the red-vented bulbul are provided. The “Effect” column indicates if presence of the bulbul affect the abundance of the species in a positive or negative way. Significant results are in bold. (DOCX) [file pone.0192249.s004.docx]

**S4 Table. Mean abundance of 14 bird species in man-modified habitats depending on the presence of the red-vented bulbul.** The “RVB overlap” column represent the percentage of the current range of the bulbul where each species is considered present. Sympatry corresponds to the number of sampling points at which the species as been recorded together with the bulbul during the same sampling session. Results of Student t tests between the abundance of 14 bird species and the presence of the red-vented bulbul are provided. The “Effect” column indicates if presence of the bulbul affect the abundance of the species in a positive or negative way. Significant results are in bold.

|  |  |  | **Bulbul absence** | | | | **Bulbul presence** | | | |  |  |  |
| --- | --- | --- | --- | --- | --- | --- | --- | --- | --- | --- | --- | --- | --- |
| Species | RVB overlap | Sympatry (n) | mean |  | se | (n) | mean |  | se | (n) | Effect | Student t | P value |
| *Acridotheres tristis*^A^ | 100% | 188 | 4,72 | ± | 0,36 | (261) | 4,4 | ± | 0,36 | (215) |  | 0,63 | 0,53 |
| *Corvus moneduloides* | 65% | 8 | 1,76 | ± | 0,16 | (42) | 0,07 | ± | 0,03 | (215) | - | **-10,47** | **1,70E-13** |
| *Gerygone flavolateralis flavolateralis* | 99% | 14 | 1,36 | ± | 0,09 | (59) | 0,11 | ± | 0,03 | (215) | - | **-12,57** | **< 2,2E-16** |
| *Lalage leucopyga montrosieri* | 99% | 9 | 1,57 | ± | 0,18 | (30) | 0,07 | ± | 0,02 | (215) | - | **-8,39** | **2,28E-09** |
| *Lichmera incana incana* | 100% | 209 | 5,8 | ± | 0,22 | (329) | 6,43 | ± | 0,29 | (215) |  | 1,73 | 0,083 |
| *Myiagra caledonica caledonica* | 96% | 5 | 1,96 | ± | 0,3 | (23) | 0,04 | ± | 0,02 | (215) | - | **-6,43** | **1,76E-06** |
| *Myzomela caledonica* | 89% | 1 | 1,81 | ± | 0,33 | (11) | 0,01 | ± | 0,01 | (215) | - | **-5,56** | **0,00024** |
| *Pachycephala rufiventris xanthetraea* | 99% | 39 | 1,83 | ± | 0,09 | (118) | 0,29 | ± | 0,05 | (215) | - | **-13,95** | **< 2,2E-16** |
| *Passer domesticus*^A^ | 87% | 158 | 7,99 | ± | 0,82 | (144) | 6,61 | ± | 0,77 | (215) |  | -1,27 | 0,21 |
| *Philemon diemenensis* | 66% | 9 | 2,1 | ± | 0,18 | (62) | 0,07 | ± | 0,03 | (215) | - | **-11,3** | **< 2,2E-16** |
| *Rhipidura albiscapa bulgeri* | 99% | 58 | 2,15 | ± | 0,12 | (181) | 0,12 | ± | 0,06 | (215) | - | **-12,43** | **< 2,2E-16** |
| *Spilopelia chinensis*^A^ | 93% | 170 | 3,01 | ± | 0,17 | (206) | 3,94 | ± | 0,28 | (215) | + | **2,84** | **0,0048** |
| *Trichoglossus haematodus* | 100% | 43 | 3,49 | ± | 0,37 | (51) | 0,59 | ± | 0,1 | (215) | - | **-7,54** | **3,58E-10** |
| *Zosterops sp* | 100% | 146 | 4,74 | ± | 0,29 | (201) | 3,98 | ± | 0,34 | (215) |  | -1,7 | 0,088 |

*^A^ Indicates alien species.*
