# Supplementary material for: The invasive Red-vented bulbul (Pycnonotus cafer) outcompetes native birds in a tropical biodiversity hotspot
Source: PLoS One. 2018 Feb 1;13(2):e0192249. doi: 10.1371/journal.pone.0192249 (PMC5794173; doi:10.1371/journal.pone.0192249)
Supplement: S5 Table — Pycaf and actri represent the abundance of Pycnonotus cafer and Acridotheres tristis respectively. sHab represents sub-habitats. Model selection and averaging was conducted with the abundance of four species as explained variables. Characteristics of models considered in the averaging are described according the four indexes: 1) K is number of degree of freedom, 2) -LL is the log-likelihood score 3) AICc is the corrected Akaike criterion score and 4) ω is the weight of each model. (DOCX) [file pone.0192249.s005.docx]

**S5 Table. Model selection and averaging on the mean abundance of four species of native New Caledonian birds**. Pycaf and actri represent the abundance of *Pycnonotus cafer* and *Acridotheres tristis* respectively. sHab represents sub-habitats. Model selection and averaging was conducted with the abundance of four species as explained variables. Characteristics of models considered in the averaging are described according the four indexes: 1) **K** is number of degree of freedom, 2) **-LL** is the log-likelihood score 3) **AICc** is the corrected Akaike criterion score and 4) **ω** is the weight of each model.

|  |  | *R. albiscapa* | | | | *Z. sp* | | | | *P.rufiventris* | | | | *T. haematodus* | | | |
| --- | --- | --- | --- | --- | --- | --- | --- | --- | --- | --- | --- | --- | --- | --- | --- | --- | --- |
|  | Models | **K** | **-LL** | **AICc** | **ω _i_** | **K** | **-LL** | **AICc** | **ω _i_** | **K** | **-LL** | **AICc** | **ω _i_** | **K** | **-LL** | **AICc** | **ω _i_** |
| Full : | pycaf + actri + year + shab | 8 | 509 | 1034,3 | 0,172 | 8 | 1126,3 | 2268,9 | 0,403 | 8 | 329,94 | 676,32 | 0,2 | - | - | - | - |
|  | pycaf + actri + shab | 7 | 509,1 | 1032,6 | 0,415 | - | - | - | - | 7 | 330,6 | 675,57 | 0,29 | - | - | - | - |
|  | pycaf + actri + year | - | - | - | - | 5 | 1129 | 2268,2 | 0,576 | - | - | - | - | 5 | 409,2 | 828,6 | 0,17 |
|  | pycaf + shab + year | - | - | - | - | - | - | - | - | 7 | 331 | 676,43 | 0,19 | - | - | - | - |
|  | pycaf + actri | - | - | - | - | - | - | - | - | - | - | - | - | 4 | 410,5 | 829,09 | 0,13 |
|  | pycaf + shab | 6 | 510,5 | 1033,2 | 0,299 | - | - | - | - | 6 | 331,5 | 675,34 | 0,32 | - | - | - | - |
|  | pycaf + year | - | - | - | - | - | - | - | - | - | - | - | - | 4 | 409,5 | 827,05 | 0,37 |
|  | pycaf | - | - | - | - | - | - | - | - | - | - | - | - | 3 | 410,6 | 827,33 | 0,32 |
